# Integrative genomics and regulomics reveal the molecular architecture of complex traits in domestic chickens

**DOI:** 10.1002/imt2.70183

**Published:** 2026-10-22

**Authors:** Wei Han, Chenghao Zhou, Yuxin You, Guohui Li, Yunfen Zhu, Yijun Tang, Tao Zeng, Huiyong Zhang, Qian Xue, Jianmei Yin, Li Chen, Xing Ju, Yixiu Jiang, Jiaqi Cai, Lu Li, Xiaofeng Zhang, Zhaoyu Lin, Jindong Ren, Lizhi Lu, Dijun Chen

**Affiliations:** ^1^ Jiangsu Institute of Poultry Science Yangzhou China; ^2^ Key Laboratory of Livestock and Poultry Resources (Poultry) Evaluation and Utilization, Ministry of Agriculture and Rural Affairs Yangzhou China; ^3^ State Key Laboratory of Pharmaceutical Biotechnology, Nanjing Drum Tower Hospital, School of Life Sciences Nanjing Nanjing University China; ^4^ Institute of Animal Husbandry and Veterinary Science, Zhejiang Academy of Agricultural Sciences Hangzhou China; ^5^ National Resource Center for Mutant Mice of China, Ministry of Education Key Laboratory of Model Animal for Disease Study, Model Animal Research Center, Nanjing Drum Tower Hospital, School of Medicine, Nanjing University Nanjing China; ^6^ Chemistry and Biomedicine Innovation Center, Nanjing University Nanjing China

## Abstract

Domestic chickens (*Gallus gallus domesticus*) exhibit exceptional phenotypic diversity and provide a valuable model for studying domestication and artificial selection. We integrated whole‐genome resequencing data from ~3000 individuals with 20 slaughter‐related traits. Population genomic analyses revealed pronounced genetic differentiation between northern and southern Chinese indigenous chickens, accompanied by distinct signatures of domestication and local adaptation. To dissect the gene regulatory networks of slaughter‐related traits, we developed an Integrated Weighted Regulatory Network Analysis (iWRNA) framework that combines genome‐wide association signals with multi‐tissue transcriptomic and epigenomic data. This approach enabled the identification of tissue‐specific regulatory modules and candidate causal genes underlying economically important traits. Notably, liver‐weight‐associated loci converged on a hepatic metabolic network in which enhancer activity coordinates gene expression and phenotypic variation. These findings establish a comprehensive regulatory atlas for complex trait variation in chickens and provide new insights into the molecular mechanisms of domestication, adaptation, and vertebrate phenotypic evolution.

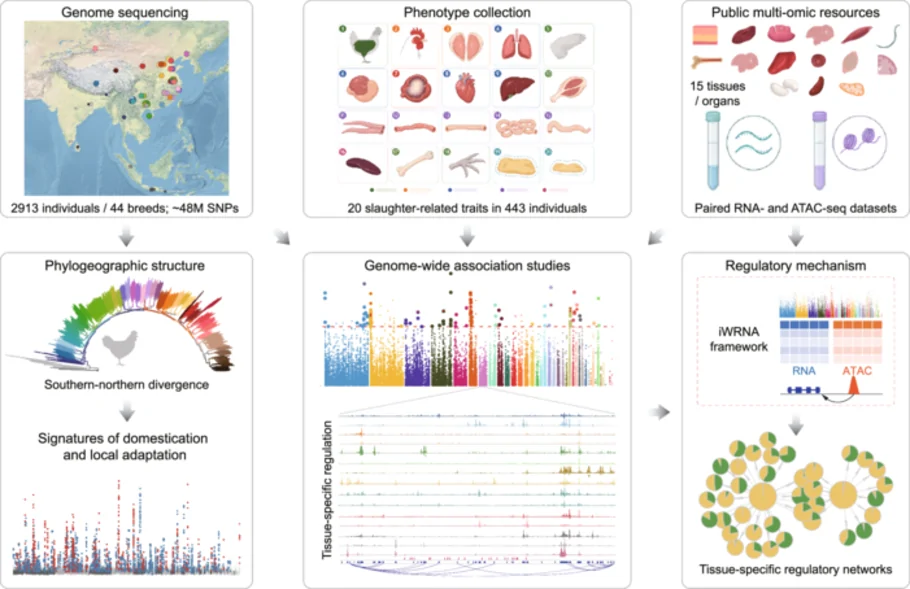

## ETHICS STATEMENT

All procedures involving chickens in this study were conducted in accordance with the relevant guidelines for the ethical review of laboratory animal welfare and were approved by the Laboratory Animal Welfare and Animal Experimentation Ethics Review Committee of Jiangsu Institute of Poultry Science (approval no. JQS2023009C).

To the Editor,

Domesticated chickens (*Gallus gallus domesticus*) are economically and scientifically important livestock, serving as a key global protein source and a model for studying complex traits shaped by artificial selection. Domestication and selective breeding over millennia have generated phenotypic diversity in growth rate, body composition, reproduction, and disease resistance [1]. These traits are controlled by intricate genetic and regulatory networks shaped by natural and artificial pressures [2]. Despite substantial progress in poultry genomics [3], the regulatory mechanisms connecting genomic variation to phenotypic diversity remain incompletely understood. Unraveling this complexity is fundamental to improving breeding efficiency and meat quality and understanding the molecular basis of domestication.

China harbors diverse chicken breeds domesticated and selected under varied ecological, cultural, and breeding contexts. These breeds range from fast‐growing commercial broilers to locally adapted or ornamental indigenous breeds and vary in morphological, physiological, and production traits [4]. Nationwide sampling captures this genetic and phenotypic diversity, enabling identification of genomic signatures associated with domestication, breed formation, and subsequent artificial or region‐specific selection. Recent livestock multi‐omics studies show that integrating population genomic, transcriptomic, and epigenomic information can help identify selection signatures and loci associated with complex traits [5].

In this study, we conducted an integrative analysis to uncover the genetic and regulatory architecture underlying agronomic traits in domestic chickens. We performed deep whole‐genome resequencing of 2913 chickens representing 44 breeds across China, encompassing diverse ecological and breeding backgrounds. This large‐scale dataset yielded approximately 48 million high‐quality genomic variants. Population structure and selection signature analyses revealed genetic differentiation among breeds and identified candidate genomic regions showing shared and region‐specific selection signatures. We performed genome‐wide association studies (GWAS) of 20 carcass and production‐related traits in 443 representative individuals, identifying multiple candidate loci associated with growth, fat deposition, and intestinal length. To explore the potential regulatory basis of these associations, we integrated ATAC‐seq and RNA‐seq data from multiple tissues, constructing a comprehensive map of chromatin accessibility and gene expression.

## A LARGE‐SCALE GENOMIC VARIATION MAP OF DOMESTIC CHICKENS

To construct a large‐scale landscape of genomic diversity in domestic chickens, we performed deep whole‐genome resequencing of 2913 individuals representing 44 breeds (each with 25 to 231 individuals) sampled across China, including local landraces, commercial breeding lines, and introduced breeds (Figures 1A, S1, and Table S1). In parallel, genomic data from 164 individuals representing red junglefowl and other junglefowl species were obtained from published studies to serve as an evolutionary reference [6] (Table S2). Many public junglefowl datasets had low sequencing depth (typically ~5×), and we leveraged our high‐coverage data (>20×) as a reference panel to impute missing genotypes (Figure S2). As expected, imputation accuracy decreased with increasing genetic divergence from the reference panel. Nevertheless, accuracy remained high (90–98%) across all red junglefowl subspecies when the target sequencing depth exceeded 2× (Figure S3).

**FIGURE 1:**
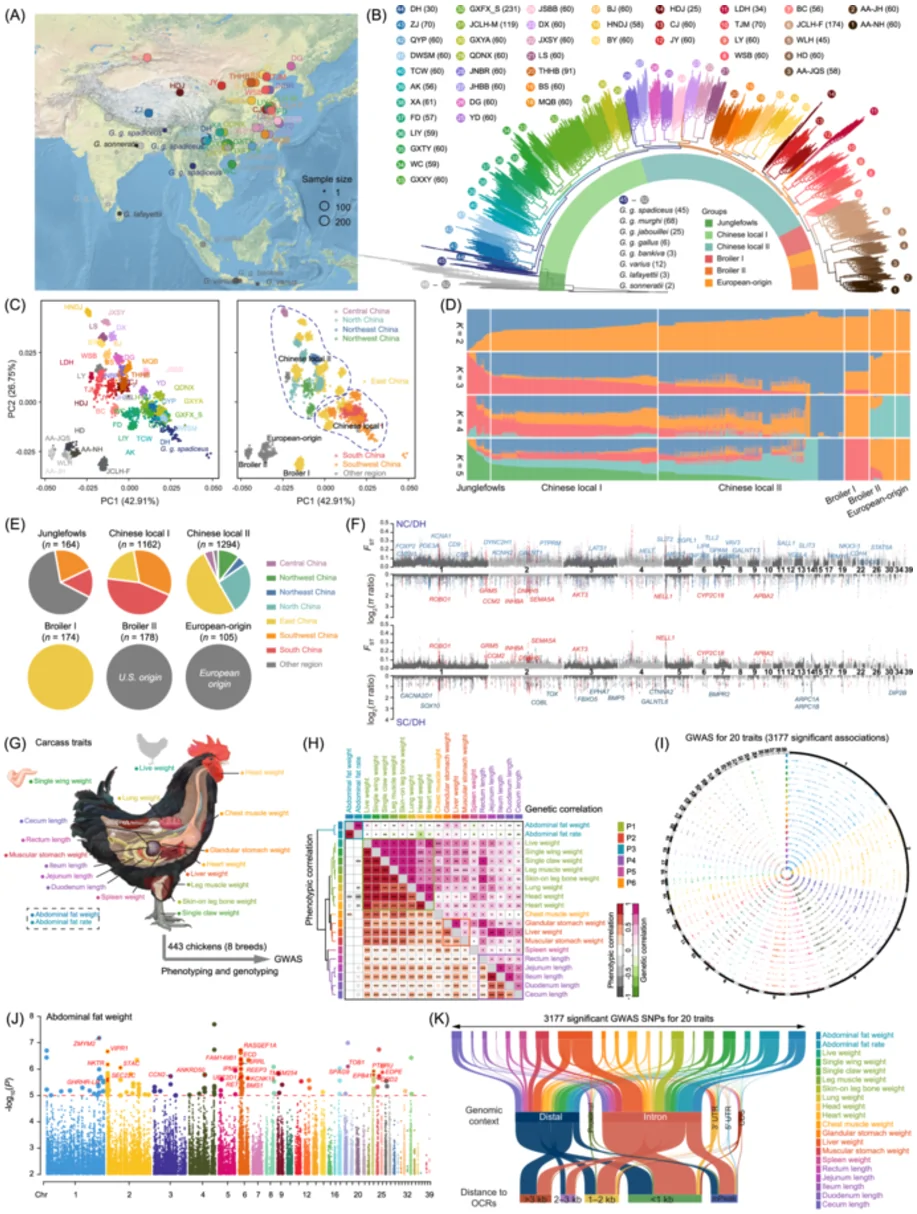
Genomic landscape, adaptive evolution, and genetic basis of complex traits in Chinese chickens. (A) Geographic distribution and breed composition of domestic chicken breeds sampled across China, including local landraces, breeding lines, and introduced breeds, alongside junglefowls as wild references. Circle size represents the number of sampled individuals. Color codes in (B) representing individual breeds are consistently used throughout the figure. (B) Maximum‐likelihood phylogenetic tree constructed from genome‐wide SNPs, showing the genetic relationships among junglefowls and domestic chicken breeds. The inner colored ring indicates the major population groups. (C) Principal component analysis (PCA) of genome‐wide SNP variation, with individuals colored by chicken breed identity (left) or geographic origin and population group (right), revealing separation between junglefowls and domestic populations and genetic structure among Chinese local chickens. (D) Ancestry inference based on ADMIXTURE analysis at *K* = 2–5. Each vertical bar represents an individual, colored according to its inferred ancestry proportions. (E) Geographic composition of the six major population groups. Pie charts show the geographic origins of Junglefowls, Chinese local chickens, Broilers, and European‐origin chickens. (F) Genome‐wide selective sweep analysis comparing northern (NC) and southern (SC) Chinese local chicken populations with Dehong chicken (DH) using *F*_ST_ and log_2_(π ratio) statistics. Shared and region‐specific candidate genes under selection are highlighted. (G) Overview of the 20 carcass‐related phenotypic traits analyzed in this study. Color codes representing individual traits are consistently used throughout the figure. (H) Phenotypic and genetic correlation matrices across all 20 carcass traits. Six major phenotypic groups were identified based on Spearman's rank correlations: body and organ mass (P1), digestive organs (P2), abdominal fat (P3), intestinal length (P4), immune organs (P5), and muscle‐related traits (P6). (I) Circos plot summarizing genome‐wide SNP–trait associations across the 20 carcass traits. The outer track represents genomic coordinates, and the inner tracks display −log_10_(*p*) values using the same vertical scale across all traits. SNPs with *p* < 1 × 10^−3^ are shown for visualization. (J) Representative Manhattan plot of GWAS results for abdominal fat weight. Candidate genes located within or near candidate association signals are annotated. (K) Genomic annotation of candidate trait–SNP associations showing their distribution across distal, promoter, intronic, untranslated, and coding regions. Over half of the associated variants overlapped with or were located near (<1 kb) open chromatin regions (OCRs) defined by ATAC‐seq.

The high sequencing depth of our dataset supported accurate calling of common and rare variants. After stringent quality control, we identified approximately 48 million high‐confidence single‐nucleotide polymorphisms (SNPs; Figure S4), representing one of the largest high‐coverage SNP catalogs of Chinese chicken populations to date and complementing global chicken reference‐panel resources [7]. Sequencing‐depth and clean‐read distributions were compared with public data [8, 9] (Figure S5).

Linkage disequilibrium (LD) decay analyses revealed a gradient associated with demographic and breeding histories. Wild junglefowl exhibited the fastest LD decay, consistent with a lower impact of domestication and intensive breeding. Chinese local landraces showed intermediate decay rates, whereas commercial broilers displayed the slowest decay, indicative of strong selection and founder effects during intensive breeding (Figure S6).

Phylogenetic reconstruction based on genome‐wide SNPs separated wild and domestic chickens, while local landraces clustered into multiple well‐supported clades, indicating genetic structure among Chinese local populations (Figure 1B). Principal component analysis (PCA) further supported these relationships, with the distribution along the first principal component broadly consistent with the phylogeny (Figure 1C).

Admixture analysis revealed mixed ancestry among domestic chickens, broadly distinguishing junglefowls, Chinese local clusters, broiler clusters, and a European‐origin lineage (Figure 1D). Broiler clusters each displayed relatively homogeneous ancestry dominated by a single ancestry component, consistent with intensive artificial selection and closed breeding. In contrast, Chinese local chickens exhibited varying degrees of mixed ancestry, including distinct wild ancestry components, consistent with historical introgression or shared ancestral variation [6]. Among Chinese local chickens, Cluster I predominantly comprised breeds sampled from southern, southwestern, and eastern China, whereas Cluster II mainly comprised breeds from eastern, northern, northeastern, and northwestern China (Figure 1E), revealing a broad north–south geographic pattern. Chickens sampled from East China were represented in both clusters, consistent with this sampling region spanning the Qinling–Huaihe Line, a recognized geographic boundary between northern and southern China [10].

## GENOME‐WIDE SIGNATURES OF SHARED AND REGION‐SPECIFIC SELECTION

To investigate the genomic basis of this north–south pattern, Chinese local landraces were independently assigned to southern and northern geographic groups based on their sampling locations relative to the Qinling–Huaihe Line. Dehong chicken (DH), an ancestral‐type indigenous breed from Yunnan that overlaps geographically with *Gallus gallus spadiceus* and shows similar morphology and close phylogenetic affinity to this subspecies [6] (Figure 1B), was used as the reference population to ensure comparable sequencing depth and consistent data processing across populations in the selection scans.

To detect putative selective sweeps, we used population differentiation (fixation index, *F*_ST_), nucleotide diversity ratio (π ratio), and cross‐population composite likelihood ratio statistics (Figure 1F; Table S3). Both comparisons revealed extensive candidate regions with selection signals. Shared sweep regions included candidate genes *AKT3, NELL1, APBA2, PCDH10, ROBO1, SEMA5A*, and *INHBA*, implicating biological processes related to growth, behavior, immunity, and reproduction (Figure 1F; Table S3).

Notably, the selective sweep regions detected in northern populations were generally larger and encompassed more genes than those in southern populations (Figure S7), indicating a more extensive genomic footprint of selection in northern populations. While shared sweeps may reflect common selective targets, region‐specific signals revealed distinct selective profiles between southern and northern Chinese chickens.

Among the southern‐specific candidates, *BMPR2, CTNNA2*, and *DIP2B* represented prominent signals, whereas northern‐specific sweep regions included *SLIT2, LIPA, FOXP2*, and *VAV3*. Among these candidates, *CTNNA2* has direct genetic evidence supporting its involvement in cortical neuronal migration [11], while *LIPA* encodes lysosomal acid lipase, a key enzyme for triglyceride and cholesteryl ester hydrolysis [12]. Collectively, these region‐specific signals suggest that selection may have differentially targeted developmental, metabolic, and neurophysiological processes in southern and northern indigenous chicken populations.

## GENOME‐WIDE ASSOCIATION ANALYSIS OF 20 CARCASS TRAITS IN DOMESTIC CHICKENS

We performed GWAS on 443 individuals representing eight domestic chicken breeds with phenotypic measurements (Table S4). Twenty slaughter‐related traits were analyzed, including carcass composition (e.g., live weight, leg and chest muscle weight, abdominal fat weight and rate, single wing weight, and head weight) and internal organ weights (e.g., heart, liver, lung, glandular and muscular stomach, and spleen weights), as well as intestinal length traits (duodenum, jejunum, ileum, cecum, and rectum length) (Figure 1G).

Phenotypic correlation analysis based on Spearman's rank coefficients revealed strong inter‐trait relationships, consistent with shared physiological and developmental influences among related traits (Figure 1H). The 20 traits formed six phenotypic groups, corresponding to body and organ mass (P1), digestive organs (P2), abdominal fat (P3), intestinal length (P4), immune organs (P5), and muscle‐related traits (P6) (Figure 1H). BOLT‐REML genetic correlations were highly concordant with phenotypic correlations (Mantel test, *r* = 0.882, *p* < 0.001; Figure S8), suggesting shared genetic contributions to the covariation among carcass traits.

GWAS across all 20 phenotypes detected 3177 significant trait–SNP associations after cluster‐based filtering (*p* < 1.0 × 10^−5^; Figure S9), ranging from 10 for jejunum length to 605 for liver weight (Figure 1I; Table S5). Most significant associations were trait‐specific, suggesting distinct genetic architectures underlying different carcass traits (Figure S10). For instance, significant variants associated with abdominal fat weight were located within or near *VIPR1, IPMK, RASGEF1A, SPAG9, STAC, CCN2*, and *TOB1* (Figure 1J), suggesting a polygenic basis for abdominal fat deposition.

Most candidate trait‐associated variants (97.8%) were noncoding, predominantly intronic or intergenic. Notably, over half of these variants (55.3%) overlapped with or were located near (<1 kb) open chromatin regions (OCRs) identified by ATAC‐seq (Figure 1K; Table S6), indicating potential involvement in *cis*‐regulatory modulation of gene expression. These findings suggest that noncoding variants may contribute to growth, metabolism, and tissue differentiation in domestic chickens.

## TRAIT‐ASSOCIATED VARIANTS REVEAL TISSUE‐SPECIFIC REGULATORY ARCHITECTURE

To investigate the potential regulatory basis of trait–SNP associations, we integrated GWAS signals with multi‐tissue epigenomic and transcriptomic datasets, including matched ATAC‐seq and RNA‐seq profiles from diverse chicken tissues [13], as well as gene expression and expression quantitative trait loci (eQTL) data from the ChickenGTEx resource [9]. Multi‐tissue epigenomic atlases in other livestock species have similarly demonstrated the value of cross‐tissue regulatory annotations for interpreting complex traits, domestication, and breeding [14]. For each candidate GWAS SNP, we identified nearby OCRs and expressed genes by genomic proximity and assessed the correlation between OCR accessibility and gene expression across tissues, thereby linking trait‐associated variants to potential regulatory elements and candidate target genes (Figure S11A). These correlations identified putative *cis*‐regulatory element (CRE)‐gene pairs with coordinated activity. The OCR–gene correlations were significantly higher than those of randomly paired OCRs and genes (*p* < 2.8 × 10^−7^, permutation test; Figure S11B,C). These genes showed pronounced tissue‐specific expression closely mirroring their corresponding OCR accessibility (Figure S11D). This strong concordance supports a model in which trait‐associated SNPs may modulate tissue‐dependent transcriptional programs by perturbing CRE activity, thereby contributing to phenotypic variation.

A spleen‐weight‐associated locus at the *APAF1* gene contained eight candidate association SNPs within the gene body, among which two distinct SNP clusters (comprising seven SNPs) overlapped with spleen‐specific OCRs (Figure S12A–C). Consistently, *APAF1* exhibited elevated expression in immune‐related organs, including the spleen, lung, bursa, and thymus (Figure S12D), in line with its established role in apoptosis and immune regulation [15]. *APAF1* harbored numerous spleen‐specific eQTLs in the ChickenGTEx dataset [9], which were organized into several co‐regulated clusters (Figure S12E,F). The second GWAS SNP cluster strongly overlapped an eQTL cluster with positive effect sizes (Figure S12F), indicating that allelic variation at these sites is associated with increased *APAF1* expression in the spleen. These findings suggest that variants within the *APAF1* locus may be associated with tissue‐selective *APAF1* expression, providing a candidate regulatory mechanism for variation in spleen weight.

A liver‐weight‐associated region between *AKR1D1* and *CGTL* harbored 22 candidate trait‐associated variants (Figure S13A,B). Both genes exhibited liver‐enriched expression in our dataset. *AKR1D1* has an established role in hepatic bile acid and metabolic regulation [16], whereas the potential contribution of *CGTL* is supported here primarily by its liver‐enriched expression and the surrounding liver‐accessible OCRs. All associated SNPs were positioned within noncoding regions densely populated by OCRs, many of which displayed markedly elevated accessibility in the liver relative to other tissues (Figure S13C). This liver‐specific chromatin accessibility pattern suggests that the associated SNPs may reside within candidate regulatory elements linked to *AKR1D1* and *CGTL* expression.

We examined GWAS results for 18 additional traits (Figures S14–S18). Across these traits, trait‐associated SNPs were preferentially located within tissue‐enriched OCRs and frequently linked to genes exhibiting concordant tissue‐specific expression patterns. Together, these findings suggest widespread links between noncoding trait‐associated variants and tissue‐specific transcriptional programs, which may contribute to complex phenotypic diversity.

## INTEGRATIVE NETWORK ANALYSIS IDENTIFIES COORDINATED GENE‐REGULATORY MODULES UNDERLYING CARCASS TRAITS

To systematically dissect the regulatory architecture underpinning complex traits, we developed a framework termed Integrated Weighted Regulatory Network Analysis (iWRNA). Related multi‐omic approaches have demonstrated that jointly modeling chromatin accessibility and gene expression can facilitate the reconstruction of cis‐regulatory dynamics and networks [17]. This framework extends the classical weighted gene co‐expression network analysis (WGCNA) algorithm to jointly model transcript co‐expression (RNA‐seq) and chromatin co‐accessibility (ATAC‐seq), enabling the identification of regulatory modules that couple *cis*‐regulatory elements with their putative target genes. We resolved 70 gene modules (g1–g70) and 105 OCR modules (p1–p105), which together reflect coordinated transcriptional and chromatin accessibility programs across tissues (Figure 2A). Integrating matched RNA‐seq and ATAC‐seq profiles further allowed the identification of OCR–gene links (|*r*| > 0.7), thereby identifying putative target genes of noncoding regulatory elements (Figure 2A).

**FIGURE 2:**
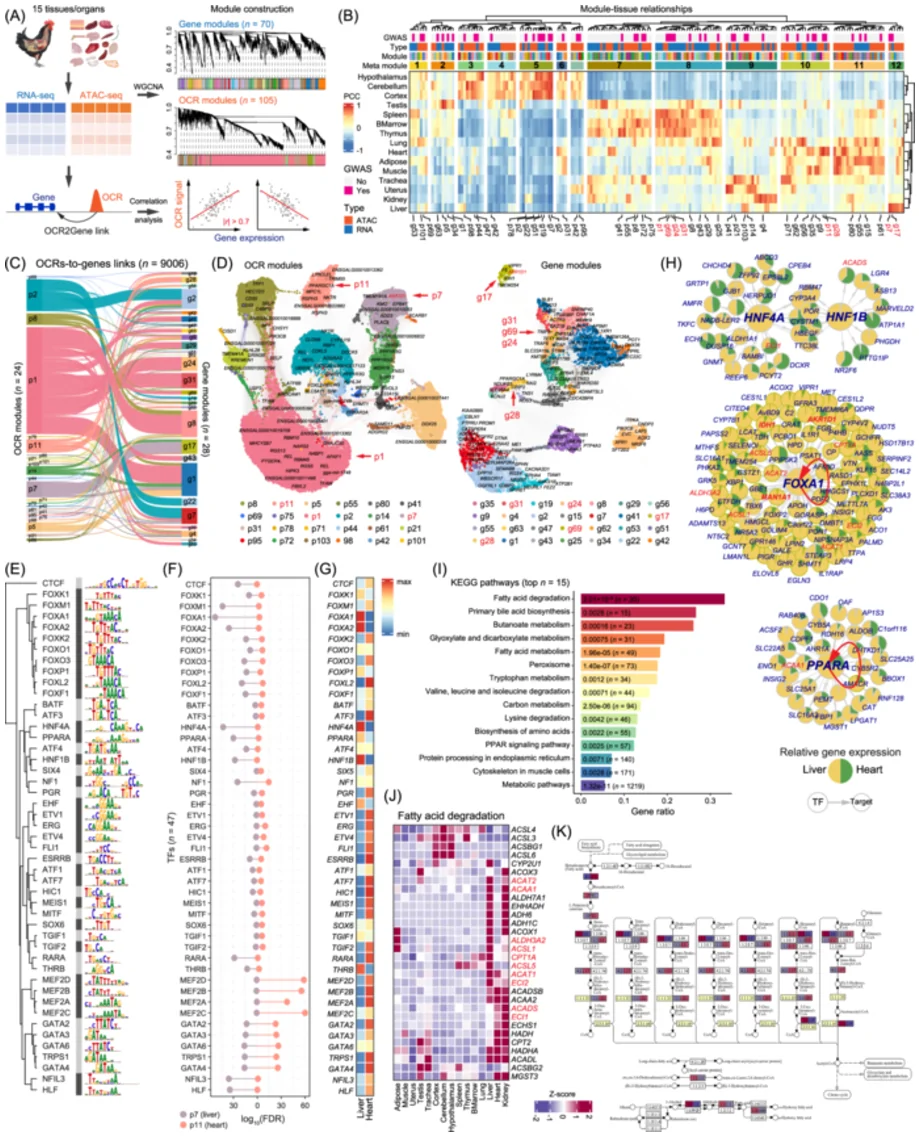
Multi‐omic dissection of regulatory modules and gene regulatory networks underlying carcass traits. (A) Schematic overview of the Integrated Weighted Regulatory Network Analysis (iWRNA) framework. Gene co‐expression modules (g1–g70) and OCR co‐accessibility modules (p1–p105) were constructed from multi‐tissue RNA‐seq and ATAC‐seq data using WGCNA. Color codes representing individual modules are consistently used throughout the figure. Significant OCR–gene correlation links (|*r*| > 0.7) were identified to predict putative regulatory relationships. (B) Heatmap of module–tissue relationships showing hierarchical clustering of 12 higher‐order meta‐modules. Each meta‐module contains gene and OCR modules with concordant tissue‐specific activity. Representative modules discussed in the main text are highlighted. (C) Sankey diagram showing relationships between gene modules and OCR modules. Connections represent 9006 significant OCR–gene links, illustrating coordinated relationships between chromatin accessibility and gene expression. (D) Topological organization of trait‐associated modules visualized by UMAP projection of the topological overlap matrix (TOM), illustrating the global network structure. Liver weight‐associated modules (g17 and p7), heart weight‐associated modules (g28 and p11), and cecum length‐associated modules (g31, g69, and p1) are highlighted. (E, F) Motif enrichment analysis of OCRs in the liver‐associated p7 module and heart‐associated p11 module, showing significantly enriched transcription factor (TF) motifs identified by HOMER. (E) Motif logos display alongside the hierarchical dendrogram of TFs based on motif similarity. (F) Enrichment scores of significantly overrepresented TF motifs in each module. (G) Relative expression profiles of the associated TFs in liver and heart, illustrating tissue‐restricted expression patterns consistent with motif enrichment. (H) Liver‐associated gene regulatory network highlighting major hub TFs, including HNF4A, HNF1B, FOXA1, and PPARA, and their candidate target genes. Each node represents a gene, with pie charts indicating its relative expression levels in liver and heart tissues. (I) KEGG pathway enrichment of genes contained in regulons controlled by major TFs. The top 15 enriched pathways are shown, with bar length representing the gene ratio. (J) Expression profiles of genes involved in the fatty acid degradation pathway across multiple tissues. Genes incorporated into the inferred gene regulatory network are highlighted in red. (K) Schematic representation of the fatty acid degradation pathway, showing the positions of genes incorporated into the inferred regulatory network. UMAP, uniform manifold approximation and projection.

To explore the functional relationships between these modules, we analyzed their correlation patterns across tissues. Hierarchical clustering based on module–tissue relationships derived from WGCNA revealed 12 higher‐order meta‐modules, each comprising tightly connected gene and OCR modules exhibiting concordant tissue‐specific activity (Figure 2B). As expected, genes and OCRs belonging to the same meta‐module were frequently connected by significant OCR–gene links (Figure 2C). For instance, in meta‐module 12, OCR module p7 and gene module g17 exhibited a strong positive correlation and highly concordant tissue‐specific enrichment profiles, both predominantly active in the liver (Figure 2B,C). Collectively, these meta‐modules represent candidate integrated units of coordinated transcriptional and chromatin activity across tissues, providing a framework for linking noncoding regulatory variation to tissue‐specific phenotypic traits.

To further investigate the potential functional relevance of these regulatory modules, we evaluated the enrichment of GWAS signals within each gene and OCR module using hypergeometric tests. Nearly one‐third of all modules (52/175, 29.7%) showed enrichment for at least one trait (nominal *p* < 0.05), comprising 41 gene module–trait pairs and 27 OCR module–trait pairs (Table S7). To assess whether these numbers exceeded random expectations, we conducted a global 1000‐iteration permutation test by randomly shuffling module assignments. The observed numbers of significant module–trait pairs were greater than expected under the corresponding randomized null distributions (empirical permutation *p* < 0.05 for each module type; Figure S19A), indicating that trait‐associated variants preferentially cluster within specific coordinated networks. Certain dual‐modules (containing gene and OCR modules) were enriched for phenotype‐associated variants: gene modules g69 and g31, together with OCR module p1, were enriched for variants linked to cecum length; modules p11 and g28 were associated with heart weight; and modules p7 and g17 were associated with liver weight (Figure S19B). Consistently, heart‐ and liver‐associated modules exhibited strong activity in heart and liver tissues, respectively (Figure S20A,B), supporting the tissue specificity of these trait‐associated modules.

To visualize trait‐associated module topology, we projected the topological overlap matrix (TOM) of the co‐expression or co‐accessibility network onto a nonlinear manifold using uniform manifold approximation and projection (UMAP), revealing module interconnections and highly connected candidate hub genes (Figure 2D). Pathway enrichment analysis revealed that trait‐associated gene modules were significantly enriched for biological processes relevant to their respective phenotypes (Figure S21). Specifically, the liver‐weight module (g17) was predominantly associated with metabolic processes, including small molecule biosynthesis and catabolism, organic and carboxylic acid metabolism, and amino acid metabolism, consistent with hepatic metabolic functions. The heart‐weight module (g28) was enriched for muscle system processes, striated muscle development, cardiocyte differentiation, and heart contraction, consistent with cardiac structural and functional processes. Finally, the cecum‐length module (g31) was linked to immune responses, cell adhesion, and T cell activation, suggesting associations with cellular processes relevant to intestinal tissue integrity, immune surveillance, and cecum development.

## TISSUE‐SPECIFIC TRANSCRIPTIONAL HIERARCHIES ASSOCIATED WITH TRAIT‐RELATED MODULES

To investigate the potential regulatory mechanisms underlying these trait‐associated modules, we performed motif enrichment analysis on OCRs within each module using HOMER. Module‐specific enrichment of transcription factor (TF) binding motifs suggested candidate TFs that may orchestrate gene expression within distinct regulatory networks (Figure S22).

Specifically, we focused on two representative OCR modules—p7 (liver‐associated) and p11 (heart‐associated)—and identified 47 candidate TFs whose motifs were significantly enriched in either module (Figure 2E,F). Motifs corresponding to FOXA1, FOXA2, HNF4A, and PPARA were enriched in the liver‐associated module (p7). Previous studies support key roles for FoxA factors and HNF4A in hepatic metabolic regulation [18, 19]. In contrast, motifs corresponding to GATA4, MEF2A‐D, and SOX6 were enriched in the heart‐associated module (p11). Recent studies support key roles for GATA‐family factors, including GATA4, in cardiac lineage specification [20]. These TF expression profiles mirrored their motif enrichment patterns: *FOXA1*, *FOXA2*, and *HNF4A* exhibited high liver‐specific expression, whereas *MEF2[A–D]*, *TRPS1*, and *GATA4* were predominantly expressed in the heart (Figure 2G). These findings suggest that tissue‐restricted TF networks may govern trait‐associated regulatory modules, linking distinct transcriptional programs to phenotypic diversity across tissues.

To further explore how these TFs may coordinate their target genes, we next integrated the TF‐motif information with gene co‐expression networks to construct gene regulatory networks (GRNs), similar to the strategy implemented in SCENIC [21]. A TF and its candidate target genes residing within the same co‐expression module were defined as a regulon. This analysis identified 43 regulons comprising 454 nodes, representing TFs and their putative downstream targets (Figure S23). For example, in the liver‐associated network, HNF4A/HNF1B, FOXA1, and PPARA were identified as candidate hub TFs connected to numerous metabolic genes involved in lipid oxidation (*CPT1A*, *ECH1*, and *ACADS*), amino acid metabolism (*GNMT*, *PHGDH*, and *PCYT2*), and redox homeostasis (*POR*, *IDH1*, and *GSTZ1*)—core processes of hepatic metabolic function (Figure 2H). Conversely, in the heart‐associated GRN, GATA4, MEF2C, MEF2D, and SOX6 were identified as candidate regulatory hubs connected to genes involved in cardiac muscle contraction (*TPM4*, *DSP*, and *SMTNL2*), sarcomere and cytoskeletal organization (*MYO1B*, *DST*, *PLEKHA7*, and *FERMT2*), and signal transduction and calcium handling (*ADCY5*, *PDE3A*, and *ORAI1*) (Figure S23).

To gain functional insights into the regulatory programs encoded by the GRN, KEGG pathway enrichment analysis was performed for the regulons associated with major TFs. The results revealed significant enrichment of metabolic and signaling pathways that are central to hepatic and muscular physiology (Figure 2I). Nearly one‐third of fatty acid degradation pathway genes were connected to TFs in the GRN, suggesting a potential role for these transcriptional circuits in hepatic metabolic regulation (Figure 2J,K). Together, these findings suggest hierarchical, tissue‐specific regulatory architectures that integrate modular TF networks with organ‐specific transcriptional programs and trait‐associated functional specializations.

In summary, this study bridges population genomics and regulatory biology by connecting genetic variation, noncoding regulatory elements, tissue‐specific regulatory programs, and complex phenotypes. Our large‐scale resource reveals pronounced genetic differentiation and identifies shared and region‐specific selection signatures among Chinese indigenous chickens, while linking trait‐associated variation to candidate regulatory elements, genes, and tissue‐specific networks. The iWRNA framework provides an interpretable strategy for prioritizing regulatory programs and generating testable hypotheses for the functional interpretation of noncoding variation. Although these regulatory relationships remain to be experimentally validated, the integrated resource and analytical framework presented here provides a foundation for functional genomics and molecular breeding in chicken. Future integration of additional variant classes, particularly structural variants and copy‐number variation, with regulatory and functional genomic data will further extend this framework and provide a more comprehensive view of the genetic architecture of complex traits.

## AI STATEMENT

ChatGPT (OpenAI; GPT‐5.6) was used to assist with language editing and formatting checks during manuscript preparation. All content has been verified for accuracy by the authors.

## AUTHOR CONTRIBUTIONS

**Wei Han**: Conceptualization; investigation; supervision; funding acquisition; project administration; resources; writing—review and editing. **Chenghao Zhou**: Investigation; writing—review and editing; data curation; resources. **Yuxin You**: Methodology; data curation; formal analysis; visualization; writing—review and editing; writing—original draft. **Guohui Li**: Data curation; investigation. **Yunfen Zhu**: Data curation; investigation. **Yijun Tang**: Data curation; investigation; visualization. **Tao Zeng**: Data curation; investigation. **Huiyong Zhang**: Data curation; investigation. **Qian Xue**: Data curation; investigation. **Jianmei Yin**: Data curation; investigation. **Li Chen**: Data curation; investigation. **Xing Ju**: Data curation; investigation. **Yixiu Jiang**: Investigation; data curation. **Jiaqi Cai**: Data curation; investigation. **Lu Li**: Data curation; investigation. **Xiaofeng Zhang**: Data curation; investigation. **Zhaoyu Lin**: Investigation; data curation. **Jindong Ren**: Investigation; data curation. **Lizhi Lu**: Conceptualization; resources; funding acquisition; project administration; supervision; writing—review and editing. **Dijun Chen**: Conceptualization; software; methodology; project administration; supervision; writing—review and editing; formal analysis; writing—original draft; visualization; funding acquisition.

## CONFLICT OF INTEREST STATEMENT

The authors declare no conflicts of interest.

## Supporting information


**Figure S1:** Representative morphological diversity of the domestic chickens sampled in this study.
**Figure S2:** Schematic workflow of reference‐panel construction and genotype imputation.
**Figure S3:** Imputation accuracy across junglefowl taxa at different downsampled sequencing depths.
**Figure S4:** Genome‐wide SNP density across 1‐Mb non‐overlapping windows.
**Figure S5:** Sequencing depth and clean‐read count distributions across datasets.
**Figure S6:** Linkage disequilibrium decay across representative chicken populations.
**Figure S7:** Comparative landscape of selective sweep regions between northern and southern populations.
**Figure S8:** Correspondence between phenotypic and genetic correlation structures.
**Figure S9:** Quantile–quantile plots of GWAS results for 20 carcass traits.
**Figure S10:** Genome‐wide association signals across 20 carcass traits.
**Figure S11:** Trait‐associated variants reveal tissue‐specific regulatory architecture.
**Figure S12:** GWAS analysis for spleen weight at the *APAF1* locus.
**Figure S13:** GWAS analysis for liver weight at the locus spanning *AKR1D1* and *CGTL*.
**Figure S14:** GWAS and locus‐level characterization for rectum length and cecum length.
**Figure S15:** GWAS and locus‐level characterization for heart weight and leg muscle weight.
**Figure S16:** GWAS and locus‐level characterization for abdominal fat rate and abdominal fat weight.
**Figure S17:** Manhattan plots for additional growth‐ and bone‐related traits.
**Figure S18:** Manhattan plots for additional intestine‐length and organ‐weight traits.
**Figure S19:** Enrichment of GWAS signals within gene and OCR modules.
**Figure S20:** Tissue‐specific activity of trait‐associated gene and OCR modules.
**Figure S21:** Gene Ontology enrichment of trait‐associated gene modules.
**Figure S22:** Motif enrichment and transcription factor activity across trait‐associated p‐modules.
**Figure S23:** Global transcription factor–target regulon network.

**Table S1:** Information on the 44 chicken breeds included in this study.
**Table S2:** Sequencing and mapping statistics of high‐depth WGS samples generated in this study and publicly available samples re‐mapped in this work.
**Table S3:** List of candidate genes putatively under selection.
**Table S4:** Phenotypic measurements of 20 carcass traits in the 443 individuals used for GWAS.
**Table S5:** Genome‐wide significant SNPs identified across 20 carcass‐related traits.
**Table S6:** Annotation of genome‐wide significant SNPs.
**Table S7:** Raw hypergeometric test *p* for GWAS signal enrichment in the 52 enriched modules.

## Data Availability

Whole‐genome sequencing data generated in this study have been deposited in the Genome Sequence Archive (GSA) at the National Genomics Data Center, China National Center for Bioinformation (https://ngdc.cncb.ac.cn/gsa/) under the accession number CRA034169 (https://ngdc.cncb.ac.cn/gsa/browse/CRA034169). Processed genotype calls, SNP annotations, and imputation reference panel are publicly available at https://biobigdata.nju.edu.cn/chicken3k/. The matched RNA‐seq and ATAC‐seq datasets were obtained from the European Nucleotide Archive (ENA) under accession number PRJEB37735 (https://ngdc.cncb.ac.cn/bioproject/browse/insdc/PRJEB37735) [13]. All scripts used for network construction and visualization are available at GitHub (https://github.com/compbioNJU/chicken-regulome). Supplementary materials (figures, tables, graphical abstract, slides, videos, Chinese translated version, and updated materials) may be found in the online DOI or iMeta Science http://www.imeta.science/.
