# Supplementary material for: Integrative genomics and regulomics reveal the molecular architecture of complex traits in domestic chickens
Source: Imeta. 2026 Oct 22;5(5):e70183. doi: 10.1002/imt2.70183 (PMC13690059; doi:10.1002/imt2.70183)
Supplement: Supplementary file 1 [file IMT2-5-e70183-s002.docx]

**Supporting information to**

***Integrative genomics and regulomics reveal the molecular architecture of complex traits in domestic chickens***

Wei Han^1,2,#,*^, Chenghao Zhou^1,2,#^, Yuxin You^3,#^, Guohui Li^1,2^, Yunfen Zhu^1,2^, Yijun Tang^3^, Tao Zeng^4^, Huiyong Zhang^1,2^, Qian Xue^1,2^, Jianmei Yin^1,2^, Li Chen^4^, Xing Ju^1,2^, Yixiu Jiang^1,2^, Jiaqi Cai^3^, Lu Li^5^, Xiaofeng Zhang^5^, Zhaoyu Lin^5^, Jindong Ren^4^, Lizhi Lu^4,*^ and Dijun Chen^3,6,*^

^1^ Jiangsu Institute of Poultry Science, Yangzhou 225125, China.

^2^ Key Laboratory of Livestock and Poultry Resources (Poultry) Evaluation and Utilization, Ministry of Agriculture and Rural Affairs, Yangzhou 225125, China.

^3^ State Key Laboratory of Pharmaceutical Biotechnology, Nanjing Drum Tower Hospital, School of Life Sciences, Nanjing University, Nanjing 210023, China.

^4^ Institute of Animal Husbandry and Veterinary Science, Zhejiang Academy of Agricultural Sciences, Hangzhou 310021, China.

^5^ National Resource Center for Mutant Mice of China, Ministry of Education Key Laboratory of Model Animal for Disease Study, Model Animal Research Center, Nanjing Drum Tower Hospital, School of Medicine, Nanjing University, Nanjing 210023, China.

^6^ Chemistry and Biomedicine Innovation Center, Nanjing University, Nanjing 210023, China.

^#^ These authors contributed equally.

^*^ Correspondence to: [hanwei830@163.com](mailto:hanwei830@163.com) (Wei Han), [lulizhibox@163.com](mailto:lulizhibox@163.com) (Lizhi Lu), or [dijunchen@nju.edu.cn](mailto:dijunchen@nju.edu.cn) (Dijun Chen)

# CONTENTS

# Supplementary Methods

# Supplementary Figures

Figure S1. Representative morphological diversity of the domestic chickens sampled in this study.

Figure S2. Schematic workflow of reference-panel construction and genotype imputation.

Figure S3. Imputation accuracy across junglefowl taxa at different downsampled sequencing depths.

Figure S4. Genome-wide SNP density across 1-Mb non-overlapping windows.

Figure S5. Sequencing depth and clean-read count distributions across datasets.

Figure S6. Linkage disequilibrium decay across representative chicken populations.

Figure S7. Comparative landscape of selective sweep regions between northern and southern populations.

Figure S8. Correspondence between phenotypic and genetic correlation structures.

Figure S9. Quantile–quantile plots of GWAS results for 20 carcass traits.

Figure S10. Genome-wide association signals across 20 carcass traits.

Figure S11. Trait-associated variants reveal tissue-specific regulatory architecture.

Figure S12. GWAS analysis for spleen weight at the *APAF1* locus.

Figure S13. GWAS analysis for liver weight at the locus spanning *AKR1D1* and *CGTL*.

Figure S14. GWAS and locus-level characterization for rectum length and cecum length.

Figure S15. GWAS and locus-level characterization for heart weight and leg muscle weight.

Figure S16. GWAS and locus-level characterization for abdominal fat rate and abdominal fat weight.

Figure S17. Manhattan plots for additional growth- and bone-related traits.

Figure S18. Manhattan plots for additional intestine-length and organ-weight traits.

Figure S19. Enrichment of GWAS signals within gene and OCR modules.

Figure S20. Tissue-specific activity of trait-associated gene and OCR modules.

Figure S21. Gene Ontology enrichment of trait-associated gene modules.

Figure S22. Motif enrichment and transcription factor activity across trait-associated p-modules.

Figure S23. Global transcription factor–target regulon network.

# Supplementary Tables (separate Excel file)

# Supplementary Methods

# Ethics statement

All procedures involving the chickens used in this study were conducted in accordance with relevant guidelines for ethical review of laboratory animal welfare and were approved by the Laboratory Animal Welfare and Animal Experimentation Ethics Review Committee of Jiangsu Institute of Poultry Science (approval no. JQS2023009C).

# Sample collection and whole-genome resequencing

A total of 2913 domestic chickens representing 44 local landraces, commercial breeding lines and introduced breeds were sampled across China, covering broad geographic and ecological regions. Genomic DNA was extracted from blood using a standard phenol-chloroform protocol. Sequencing libraries were performed by using BGI Optimal DNA Library Prep Kit (BGI-Shenzhen,China) and sequenced on DNBSEQ-T7 platforms to generate paired-end 150 bp reads, targeting an average coverage of 20× per individual. Additionally, 164 junglefowls were included as wild references using publicly available datasets (<http://bigd.big.ac.cn/chickensd/>).

# Variant detection, genotype calling and haplotype phasing

Raw reads were filtered using fastp [1] (v0.20.0) to remove adapters and low-quality bases (Q < 20). Clean reads were mapped to the *Gallus gallus* reference genome (GRCg7b) with BWA-MEM [2] (v0.7.15), followed by duplicate removal using Picard tools (v2.26.10). Variant calling was performed with GATK [3] (v4.3.0) HaplotypeCaller in gVCF mode, and joint genotyping across all samples was conducted using GATK GenomicsDBImport and GenotypeGVCFs. Variants were hard filtered using GATK VariantFiltration with the criteria QD < 2.0, FS > 60.0, MQ < 40.0, MQRankSum < –12.5, and ReadPosRankSum < –8.0.

Genotype phasing was performed using the SHAPEIT5 software suite following the recommended workflow [4]. Variants showing strong deviations from Hardy-Weinberg equilibrium (HWE *p*-value < 1 × 10⁻³⁰) were further removed. Genomic chunks were first generated with GLIMPSE2_chunk [5], and common variants (MAF ≥ 0.001) were phased using SHAPEIT5_phase_common with chromosome-specific recombination maps. For long chromosomes, chunk-wise phased segments were merged using SHAPEIT5_ligate. Rare variants were then refined using SHAPEIT5_phase_rare, with the ligated common-variant scaffold supplied as the reference. Finally, all phased chunks were concatenated using bcftools [6] (v1.9) concat and indexed for downstream imputation analyses.

# Imputation and accuracy evaluation

Genotype imputation was conducted using the GLIMPSE2 framework [5]. For each low-depth sample, genotype likelihoods (GLs) were computed from the BAM files using bcftools mpileup restricted to variant sites in the reference panel, and the resulting GLs were then phased using GLIMPSE2_phase. To assess imputation performance, six high-depth junglefowls (>20×) were removed from the reference panel and then downsampled to a series of depths (0.1×–4×). Accuracy was quantified by comparing the GP-filtered imputed genotypes with the original high-depth genotypes of the same individuals. Concordance was computed following the method used in the domestic goat ancient genome imputation study [7].

# Population genomic analyses

All population genomic analyses were based on VCF files that were filtered according to the recommended filtering guidelines [8]. Nucleotide diversity (π) and pairwise fixation index (F_ST_) were calculated using VCFtools [9] (v0.1.16) in 20 kb windows with a 4 kb step. Linkage disequilibrium (LD) decay was estimated using PopLDdecay [10]. Principal component analysis (PCA) was performed using PLINK [11] (v2.0) based on LD-pruned SNPs. Phylogenetic relationships were conducted using IQ-TREE [12] under the MFP+ASC model with 1000 ultrafast bootstraps. Admixture analysis was performed using ADMIXTURE [13] (v1.3.0) for K = 2–10 ancestral components, and the optimal K was selected based on cross-validation error.

# Genome-wide selection scans

To identify genomic regions under selection, we computed fixation index (F_ST_), nucleotide diversity ratio (π_wild_/π_domestic_), and cross-population composite likelihood ratio (XP-CLR) statistics between domestic populations (northern and southern) and Dehong chicken. F_ST_ and π values were calculated in 20 kb windows with a 4 kb step using VCFtools [9], while XP-CLR analysis was performed with XP-CLR [14] (v1.1; with parameters of window size = 20 kb, step size = 4 kb, maximum SNPs per window = 200). Windows falling within the top 5% of all three statistics were defined as candidate selective sweeps. Genes located within these regions were annotated using Ensembl gene annotation (GRCg7b) and used for downstream functional enrichment analyses.

# Phenotypic measurements and trait definitions

Twenty carcass-related traits were recorded in 443 representative individuals from multiple breeds, comprising live weight, 13 body/organ/tissue weight traits (heart, liver, spleen, lung, glandular stomach, muscular stomach, chest muscle, leg muscle, single wing, single claw, skin-on leg bone, abdominal fat and head weight), abdominal fat rate (abdominal fat weight divided by the sum of eviscerated carcass weight and abdominal fat weight), and five intestinal length traits (duodenum, jejunum, ileum, cecum and rectum length). All birds were slaughtered at approximately 120 days of age following a standardized slaughter and dissection protocol. Phenotypic correlations were computed by Spearman’s rank coefficients, and genetic correlations were estimated via the restricted maximum likelihood (REML) method implemented in GCTA [15] (v1.94).

# Genome-wide association studies (GWAS)

Genome-wide association analyses were performed for each trait using a linear mixed model in GEMMA [16] (v0.98.5), incorporating a centered relatedness matrix to correct for population structure. The model used was:

$$y=W\alpha+x\beta+u+\epsilon,$$

where y is the phenotype vector, W is the matrix of covariates (including the intercept, sex, batch, and the first three PCs), *α* is the vector of corresponding fixed-effect coefficients, x is the genotype vector, β is the SNP effect, u is the random effect with variance σ_g²_K, and ε is the residual error.

Candidate association SNPs were identified using a predefined screening threshold of *p*-value < 1 × 10⁻^5^. Q–Q plots and genomic inflation factors (λGC) were used to evaluate the calibration of the association tests. For each trait, genomic regions containing candidate association signals were further refined by clustering candidate association SNPs. Specifically, SNPs meeting the predefined screening threshold (*p*-value < 1 × 10⁻^5^) were first sorted by genomic position and merged into association clusters when the distance between adjacent SNPs was ≤50 kb. To reduce spurious signals, only clusters containing at least two candidate association SNPs were retained and considered candidate association regions. Within each retained cluster, the SNP with the smallest *p*-value was defined as the lead SNP. Candidate genes were subsequently defined as the nearest annotated genes (≤50 kb) to the lead SNPs within each retained cluster.

# ATAC-seq data processing and analysis

Matched transcriptome (RNA-seq) and chromatin accessibility (ATAC-seq) datasets from 15 adult chicken tissues were obtained from Pan et al [17]. ATAC-seq data were processed following the standardized workflow described in our previous studies [18−20]. Briefly, raw paired-end reads were trimmed using Trimmomatic [21] (v0.39) to remove sequencing adapters and low-quality bases. The cleaned reads were then aligned to the *Gallus gallus* reference genome (GRCg7b) using Bowtie2 [22] (v2.4.5) with parameters --very-sensitive -X 2000 --no-discordant --no-mixed. Reads mapping to mitochondrial DNA and unplaced scaffolds were excluded. Only properly paired reads with a mapping quality (MAPQ) > 30 were retained using SAMtools (v1.15), and PCR duplicates were removed with the MarkDuplicates function in Picard.

Open chromatin peaks were identified with MACS2 [23] (v2.2.7.1) using parameters “--nomodel --shift -100 --extsize 200 -B --SPMR --call-summits”. Peaks with FDR < 0.01 were retained. Peak quality and signal-to-noise ratios were assessed using the phantompeakqualtools package. Consensus open chromatin regions (OCRs) were generated by merging reproducible peaks (present in ≥2 biological replicates) with BEDTools [24] (v2.30.0). Normalized accessibility matrices (counts per million) were used for downstream analyses including co-accessibility network construction (WGCNA) and motif enrichment.

# RNA-seq data processing and expression quantification

RNA-seq data from the same 15 chicken tissues were obtained from Pan et al [17] and were quality trimmed using Trimmomatic (v0.39) and aligned to the *Gallus gallus* genome (GRCg7b) with STAR [25] (v2.7.10a) using default two-pass mode. Gene-level expression quantification was performed with RSEM [26] (v1.3.3). Expression values were normalized as transcripts per million (TPM).

To further validate tissue-specific expression patterns of candidate genes, we additionally incorporated RNA-seq data from diverse chicken tissues generated by the ChickenGTEx consortium [27] (<https://ngdc.cncb.ac.cn/chickengtex/>). Because ChickenGTEx was annotated using the *Gallus gallus* GRCg6a genome whereas our study was mapped to GRCg7b, gene identifiers between the two genome versions were harmonized using the Ensembl BioMart online tool. Only genes with unambiguous one-to-one mappings were retained for downstream comparative analyses.

# Integrated Weighted Regulatory Network Analysis (iWRNA)

To reconstruct regulatory modules linking cis-regulatory elements to their putative target genes, we developed an Integrated Weighted Regulatory Network Analysis (iWRNA) framework. This framework adapts the weighted gene co-expression network analysis (WGCNA) algorithm [28] to jointly model co-expression (RNA-seq) and co-accessibility (ATAC-seq) patterns across tissues.

For each dataset, normalized expression or accessibility matrices were used to calculate pairwise Pearson correlations, which were subsequently transformed into adjacency matrices using a soft-thresholding power (β = 8 for RNA-seq and β = 10 for ATAC-seq) to approximate scale-free topology. Modules were defined by dynamic tree cutting (minModuleSize = 30, mergeCutHeight = 0.15), yielding 70 gene modules (g1-g70) and 105 OCR modules (p1-p105). Module eigengenes were correlated across tissues to identify higher-order meta-modules, representing integrated transcriptional–chromatin units.

To associate regulatory modules with phenotypic variation, genome-wide association study (GWAS) loci were tested for enrichment within module regions using hypergeometric tests. Modules with a nominal *p*-value < 0.05 were defined as trait-associated modules. To account for potential false positives arising from multiple testing across all module–trait combinations, we performed a global permutation test (B=1000 iterations) by randomly shuffling gene and OCR assignments across modules while preserving individual module sizes, trait-associated locus counts, and genomic background sizes. The empirical *p*-value was calculated by comparing the observed number of significant pairs against the permutation null distribution to confirm the overall statistical significance of the module-trait associations. OCR–gene regulatory links (|*r*| > 0.7) were computed using our cisDynet pipeline [19], which integrates chromatin accessibility and gene expression correlations across matched tissues. These links were then mapped between corresponding co-accessibility and co-expression modules to construct composite cis-regulatory networks connecting noncoding elements to their putative target genes. Topological overlap matrices (TOMs) were visualized using UMAP, and hub genes were defined as nodes with high intramodular connectivity (kME > 0.85).

# Motif and transcription factor analysis

For trait-associated OCR modules, enriched transcription factor (TF) binding motifs were identified using HOMER [29] (v4.11; the findMotifsGenome.pl program) against a GC-matched background. Motif similarity was assessed using motifStack to cluster TFs into families. TF-gene regulatory networks were constructed by linking TFs to genes within modules containing their predicted motifs.

# Functional and pathway enrichment

Functional enrichment was performed using clusterProfiler [30] (v4.10.1) with Gene Ontology (GO) and KEGG pathway databases. Organ-specific pathways (e.g., fatty acid degradation in liver) were visualized with Pathview [31] (v1.42.0).

# Data visualization

All plots were generated using ggplot2, ComplexHeatmap [32], and ggtree [33]. Genome tracks were visualized with WU Epigenome Browser [34]. Selected graphical elements used in the schematic illustrations were sourced from BioRender.com.

# References

1.	Chen, Shifu, Yanqing Zhou, Yaru Chen, Jia Gu. 2018. “fastp: An ultra-fast all-in-one FASTQ preprocessor.” *Bioinformatics* 34: i884–90. https://doi.org/10.1093/bioinformatics/bty560

2.	Li, Heng. 2013. “Aligning sequence reads, clone sequences and assembly contigs with BWA-MEM.” *arXiv*, version 2. https://doi.org/10.48550/arXiv.1303.3997

3.	Poplin, Ryan, Valentin Ruano-Rubio, Mark A. DePristo, Tim J. Fennell, Mauricio O. Carneiro, Geraldine A. Van der Auwera, David E. Kling, et al. 2017. “Scaling accurate genetic variant discovery to tens of thousands of samples.” *bioRxiv:* 201178. https://doi.org/10.1101/201178

4.	Hofmeister, Robin J., Diogo M. Ribeiro, Simone Rubinacci, Olivier Delaneau. 2023. “Accurate rare variant phasing of whole-genome and whole-exome sequencing data in the UK Biobank.” *Nature Genetics* 55: 1243–49. https://doi.org/10.1038/s41588-023-01415-w

5.	Rubinacci, Simone, Diogo M. Ribeiro, Robin J. Hofmeister, Olivier Delaneau. 2021. “Efficient phasing and imputation of low-coverage sequencing data using large reference panels.” *Nature Genetics* 53: 120–26. https://doi.org/10.1038/s41588-020-00756-0

6.	Danecek, Petr, James K. Bonfield, Jennifer Liddle, John Marshall, Valeriu Ohan, Martin O. Pollard, Andrew Whitwham, et al. 2021. “Twelve years of SAMtools and BCFtools.” *GigaScience* 10: giab008. https://doi.org/10.1093/gigascience/giab008

7.	Erven, Jolijn A. M., Alice Etourneau, Marjan Mashkour, Mahesh Neupane, Phillipe Bardou, Alessandra Stella, Andrea Talenti, et al. 2025. “Inferring domestic goat demographic history through ancient genome imputation.” *Genome Biology and Evolution* 17: evaf181. https://doi.org/10.1093/gbe/evaf181

8.	Hemstrom, William, Jared A. Grummer, Gordon Luikart, Mark R. Christie. 2024. “Next-generation data filtering in the genomics era.” *Nature Reviews Genetics* 25: 750–67. https://doi.org/10.1038/s41576-024-00738-6

9.	Danecek, Petr, Adam Auton, Goncalo Abecasis, Cornelis A. Albers, Eric Banks, Mark A. DePristo, Robert E. Handsaker, et al. 2011. “The variant call format and VCFtools.” *Bioinformatics* 27: 2156–58. https://doi.org/10.1093/bioinformatics/btr330

10.	Zhang, Chi, Shan-Shan Dong, Jun-Yang Xu, Wei-Ming He, Tie-Lin Yang. 2019. “PopLDdecay: A fast and effective tool for linkage disequilibrium decay analysis based on variant call format files.” *Bioinformatics* 35: 1786–88. https://doi.org/10.1093/bioinformatics/bty875

11.	Chang, Christopher C., Carson C. Chow, Laurent C. A. M. Tellier, Shashaank Vattikuti, Shaun M. Purcell, James J. Lee. 2015. “Second-generation PLINK: Rising to the challenge of larger and richer datasets.” *GigaScience* 4: 7. https://doi.org/10.1186/s13742-015-0047-8

12.	Nguyen, Lam-Tung, Heiko A. Schmidt, Arndt von Haeseler, Bui Quang Minh. 2015. “IQ-TREE: A fast and effective stochastic algorithm for estimating maximum-likelihood phylogenies.” *Molecular Biology and Evolution* 32: 268–74. https://doi.org/10.1093/molbev/msu300

13.	Alexander, David H., John Novembre, Kenneth Lange. 2009. “Fast model-based estimation of ancestry in unrelated individuals.” *Genome Research* 19: 1655–64. https://doi.org/10.1101/gr.094052.109

14.	Chen, Hua, Nick Patterson, David Reich. 2010. “Population differentiation as a test for selective sweeps.” *Genome Research* 20: 393–402. https://doi.org/10.1101/gr.100545.109

15.	Yang, Jian, S. Hong Lee, Michael E. Goddard, Peter M. Visscher. 2011. “GCTA: A tool for Genome-wide Complex Trait Analysis.” *The American Journal of Human Genetics* 88: 76–82. https://doi.org/10.1016/j.ajhg.2010.11.011

16.	Zhou, Xiang, Matthew Stephens. 2012. “Genome-wide efficient mixed-model analysis for association studies.” *Nature Genetics* 44: 821–24. https://doi.org/10.1038/ng.2310

17.	Pan, Zhangyuan, Ying Wang, Mingshan Wang, Yuzhe Wang, Xiaoning Zhu, Shenwen Gu, Conghao Zhong, et al. 2023. “An atlas of regulatory elements in chicken: A resource for chicken genetics and genomics.” *Science Advances* 9: eade1204. https://doi.org/10.1126/sciadv.ade1204

18.	Zhu, Tao, Chunjiao Xia, Ranran Yu, Xinkai Zhou, Xingbing Xu, Lin Wang, Zhanxiang Zong, et al. 2024. “Comprehensive mapping and modelling of the rice regulome landscape unveils the regulatory architecture underlying complex traits.” *Nature Communications* 15: 6562. https://doi.org/10.1038/s41467-024-50787-y

19.	Zhu, Tao, Xinkai Zhou, Yuxin You, Lin Wang, Zhaohui He, Dijun Chen. 2023. “cisDynet: An integrated platform for modeling gene‐regulatory dynamics and networks.” *iMeta* 2: e152. https://doi.org/10.1002/imt2.152

20.	Fu, Liang-Yu, Tao Zhu, Xinkai Zhou, Ranran Yu, Zhaohui He, Peijing Zhang, Zhigui Wu, Ming Chen, Kerstin Kaufmann, Dijun Chen. 2022. “ChIP-Hub provides an integrative platform for exploring plant regulome.” *Nature Communications* 13: 3413. https://doi.org/10.1038/s41467-022-30770-1

21.	Bolger, Anthony M., Marc Lohse, Bjoern Usadel. 2014. “Trimmomatic: A flexible trimmer for Illumina sequence data.” *Bioinformatics* 30: 2114–20. https://doi.org/10.1093/bioinformatics/btu170

22.	Langmead, Ben, Steven L. Salzberg. 2012. “Fast gapped-read alignment with Bowtie 2.” *Nature Methods* 9: 357–59. https://doi.org/10.1038/nmeth.1923

23.	Zhang, Yong, Tao Liu, Clifford A. Meyer, Jérôme Eeckhoute, David S. Johnson, Bradley E. Bernstein, Chad Nusbaum, et al. 2008. “Model-based analysis of ChIP-Seq (MACS).” *Genome Biology* 9: R137. https://doi.org/10.1186/gb-2008-9-9-r137

24.	Quinlan, Aaron R., Ira M. Hall. 2010. “BEDTools: a flexible suite of utilities for comparing genomic features.” *Bioinformatics* 26: 841–42. https://doi.org/10.1093/bioinformatics/btq033

25.	Dobin, Alexander, Carrie A. Davis, Felix Schlesinger, Jorg Drenkow, Chris Zaleski, Sonali Jha, Philippe Batut, Mark Chaisson, Thomas R. Gingeras. 2013. “STAR: Ultrafast universal RNA-seq aligner.” *Bioinformatics* 29: 15–21. https://doi.org/10.1093/bioinformatics/bts635

26.	Li, Bo, Colin N. Dewey. 2011. “RSEM: Accurate transcript quantification from RNA-Seq data with or without a reference genome.” *BMC Bioinformatics* 12: 323. https://doi.org/10.1186/1471-2105-12-323

27.	Guan, Dailu, Zhonghao Bai, Xiaoning Zhu, Conghao Zhong, Yali Hou, Di Zhu, The ChickenGTEx Consortium, et al. 2025. “Genetic regulation of gene expression across multiple tissues in chickens.” *Nature Genetics* 57: 1298–1308. https://doi.org/10.1038/s41588-025-02155-9

28.	Langfelder, Peter, Steve Horvath. 2008. “WGCNA: An R package for weighted correlation network analysis.” *BMC Bioinformatics* 9: 559. https://doi.org/10.1186/1471-2105-9-559

29.	Heinz, Sven, Christopher Benner, Nathanael Spann, Eric Bertolino, Yin C. Lin, Peter Laslo, Jason X. Cheng, Cornelis Murre, Harinder Singh, Christopher K. Glass. 2010. “Simple combinations of lineage-determining transcription factors prime cis-Regulatory elements required for macrophage and B cell identities.” *Molecular Cell* 38: 576–89. https://doi.org/10.1016/j.molcel.2010.05.004

30.	Wu, Tianzhi, Erqiang Hu, Shuangbin Xu, Meijun Chen, Pingfan Guo, Zehan Dai, Tingze Feng, et al. 2021. “clusterProfiler 4.0: A universal enrichment tool for interpreting omics data.” *The Innovation* 2: 100141. https://doi.org/10.1016/j.xinn.2021.100141

31.	Luo, Weijun, Cory Brouwer. 2013. “Pathview: An R/Bioconductor package for pathway-based data integration and visualization.” *Bioinformatics* 29: 1830–31. https://doi.org/10.1093/bioinformatics/btt285

32.	Gu, Zuguang. 2022. “Complex heatmap visualization.” *iMeta* 1: e43. https://doi.org/10.1002/imt2.43

33.	Xu, Shuangbin, Lin Li, Xiao Luo, Meijun Chen, Wenli Tang, Li Zhan, Zehan Dai, Tommy T. Lam, Yi Guan, Guangchuang Yu. 2022. “*Ggtree*: A serialized data object for visualization of a phylogenetic tree and annotation data.” *iMeta* 1: e56. https://doi.org/10.1002/imt2.56

34.	Seng, Chanrung, Shane Liu, Wenjin Zhang, Xiaoyu Zhuo, Daofeng Li, Ting Wang. 2025. “WashU Epigenome Browser update 2025.” *Nucleic Acids Research* 53: W554–61. https://doi.org/10.1093/nar/gkaf387

# Supplementary Figures

**Figure S1.** Representative morphological diversity of the domestic chickens sampled in this study. Photographs illustrate the broad morphological diversity across populations, corresponding to their geographic origins and breeding histories. Breed abbreviations and sampling locations (province or country codes) are indicated below each image. Colored sidebars indicate ancestry groups, whereas colored dots denote breed categories (introduced breed, breeding line, or landrace).

**Figure S2.** Schematic workflow of reference-panel construction and genotype imputation. High-depth (>20×) whole-genome sequencing (WGS) samples (*n* = 2867) were used to construct the reference panel. Genotypes in 210 lower-depth samples (10–15× for samples generated in this study and approximately 5× for publicly available samples) were subsequently imputed using this panel, yielding a final dataset of 3077 chickens.

**Figure S3.** Imputation accuracy across junglefowl taxa at different downsampled sequencing depths. Imputation accuracy was evaluated at downsampled sequencing depths from 0.1× to 4×. Each row represents a different junglefowl taxon, columns represent minor allele frequency (MAF) cutoffs, and lines represent genotype probability (GP) thresholds. Accuracy was calculated as the concordance between imputed genotypes and the corresponding high-depth ground truth.

**Figure S4.** Genome-wide SNP density across 1-Mb non-overlapping windows. Genome-wide SNP density along each chromosome was calculated in 1-Mb non-overlapping windows after hard filtering. The horizontal axis represents genomic position (Mb), and color intensity indicates the number of SNPs per window.

**Figure S5.** Sequencing depth and clean-read count distributions across datasets. Distributions of (A) sequencing depth and (B) clean-read counts for samples generated in this study (*n* = 2913), together with the publicly available ChickenGTEx (*n* = 2869) and *Scientific Data* 2024 (*n* = 209) datasets.

**Figure S6.** Linkage disequilibrium decay across representative chicken populations. Linkage disequilibrium decay curves are shown separately for Broiler I, Broiler II, Chinese local landraces, and junglefowls. Linkage disequilibrium was measured as pairwise *r*² and plotted against the physical distance (kb) between SNPs.

**Figure S7.** Comparative landscape of selective sweep regions between northern and southern populations. (A) Total genomic length of candidate selective sweep regions identified in northern chickens (NC), southern chickens (SC), and regions shared between the two populations. (B) Numbers of genes associated with the corresponding candidate selective sweep regions.

**Figure S8.** Correspondence between phenotypic and genetic correlation structures. Hierarchical clustering based on phenotypic (left) and genetic (right) correlation matrices across 20 carcass traits. Lines connect the same traits between the two dendrograms. The phenotypic Spearman correlation matrix and the REML-estimated genetic correlation matrix were ordered by the same traits and converted to distance matrices using *D* = 1 − *r*. Correspondence between these distance matrices was assessed using a Pearson-based Mantel test with 999 unrestricted permutations (Mantel *r* = 0.882, *p* ≤ 0.001).

**Figure S9.** Quantile–quantile plots of GWAS results for 20 carcass traits. Quantile–quantile plots compare the observed and expected −log₁₀(*p*) values for each trait. The red diagonal line represents the expected distribution under the null hypothesis, and the genomic inflation factor (λGC) is shown in each panel.

**Figure S10.** Genome-wide association signals across 20 carcass traits. (A) Heatmap of −log₁₀(*p*) values for SNPs significant for at least one of the 20 traits. (B) Binary significance map indicating the SNP–trait associations that exceed the prespecified significance threshold (*p* < 1 × 10⁻⁵).

**Figure S11.** Trait-associated variants reveal tissue-specific regulatory architecture. (A) Overview of the integrative analysis linking GWAS SNPs to putative regulatory elements (OCRs) and target genes using multi-tissue RNA-seq and ATAC-seq data. For each significant GWAS SNP, nearby OCRs and expressed genes were identified on the basis of genomic proximity, and correlations between OCR accessibility and gene expression across tissues were subsequently calculated. (B) OCR–gene correlation analysis. Pearson correlation coefficients for observed OCR–gene pairs were significantly higher than those for randomly paired OCRs and genes (*p* < 2.8 × 10⁻⁷, permutation test). (C) OCR–gene correlations calculated separately for each trait, using randomly paired OCRs and genes from across the genome as a control (*n* = 1220). Observed OCR–gene correlations were higher than those for the random control across all traits. (D) Heatmaps of gene expression (RNA-seq; left) and chromatin accessibility (ATAC-seq; right) for SNP-associated OCR–gene pairs across tissues, illustrating coordinated tissue-dependent regulatory activity. For box plots, center lines indicate medians, boxes indicate the interquartile range, and whiskers extend to 1.5 times the interquartile range.

**Figure S12.** GWAS analysis for spleen weight at the *APAF1* locus. (A) Manhattan plot of SNP associations with spleen weight. The red dashed line indicates the significance threshold (*p* < 1 × 10⁻⁵). (B) Regional association plot at *APAF1*, with variants colored according to linkage disequilibrium (*r*²) with the lead SNP. (C) Genomic tracks at *APAF1* showing ATAC-seq signals across tissues, with significant GWAS SNPs marked by triangles. Two SNP clusters overlapping spleen-specific OCRs are highlighted. (D) *APAF1* expression across tissues based on ChickenGTEx data, with spleen-enriched expression highlighted. (E) Tissue distribution of ChickenGTEx eQTLs for *APAF1*. (F) Relationship between spleen-weight GWAS variants and spleen eQTLs at *APAF1*. Positive- and negative-effect eQTLs are shown above, and their reference- and alternative-allele frequencies are shown below. For box plots, center lines indicate medians, boxes indicate the interquartile range, and whiskers extend to 1.5 times the interquartile range.

**Figure S13.** GWAS analysis for liver weight at the locus spanning *AKR1D1* and *CGTL*. (A) Manhattan plot of SNP associations with liver weight. The red dashed line indicates the significance threshold (*p* < 1 × 10⁻⁵). (B) Genomic tracks showing ATAC-seq signals across tissues (left) and expression patterns of *AKR1D1* and *CGTL* (right), highlighting liver-enriched regulatory activity and expression. (C) Distribution of chromatin accessibility across tissues for the 20 OCRs within the *AKR1D1–CGTL* region. For box plots, center lines indicate medians, boxes indicate the interquartile range, and whiskers extend to 1.5 times the interquartile range.

**Figure S14.** GWAS and locus-level characterization for rectum length and cecum length. (A,B) Manhattan plots of SNP associations with rectum length (A) and cecum length (B). The red dashed line indicates the significance threshold (*p* < 1 × 10⁻⁵). (C,D) Regional association plots at the lead loci *SEMA5B* (C) and *NMNAT3* (D), with variants colored according to linkage disequilibrium (*r*²) with the lead SNP. (E,F) Tissue expression profiles of *SEMA5B* (E) and *NMNAT3* (F) based on ChickenGTEx data. For box plots, center lines indicate medians, boxes indicate the interquartile range, and whiskers extend to 1.5 times the interquartile range.

**Figure S15.** GWAS and locus-level characterization for heart weight and leg muscle weight. (A,B) Manhattan plots of SNP associations with heart weight (A) and leg muscle weight (B). The red dashed line indicates the significance threshold (*p* < 1 × 10⁻⁵). (C,D) Regional association plots at the lead loci *SMPX* (C) and *CACNA2D1* (D), with variants colored according to linkage disequilibrium (*r*²) with the lead SNP. (E,F) Tissue expression profiles of *SMPX* (E) and *CACNA2D1* (F) based on ChickenGTEx data. For box plots, center lines indicate medians, boxes indicate the interquartile range, and whiskers extend to 1.5 times the interquartile range.

**Figure S16.** GWAS and locus-level characterization for abdominal fat rate and abdominal fat weight. (A,B) Manhattan plots of SNP associations with abdominal fat rate (A) and abdominal fat weight (B). The red dashed line indicates the significance threshold (*p* < 1 × 10⁻⁵). (C) Genomic tracks showing ATAC-seq signals across tissues (left) and expression patterns of *DCN* and *LUM* (right). (D) Regional association plot at the lead *RET* locus, with variants colored according to linkage disequilibrium (*r*²) with the lead SNP. (E) Distribution of chromatin accessibility across tissues for the 20 OCRs within the *DCN–LUM* region. (F) Tissue expression profile of *RET* based on ChickenGTEx data. For box plots, center lines indicate medians, boxes indicate the interquartile range, and whiskers extend to 1.5 times the interquartile range.

**Figure S17.** Manhattan plots for additional growth- and bone-related traits. Growth and muscle traits: (A) live weight and (B) chest muscle weight. Bone-related traits: (C) single wing weight, (D) skin-on leg bone weight, (E) head weight, and (F) single claw weight. The red dashed line indicates the significance threshold (*p* < 1 × 10⁻⁵), and candidate genes at significant signals are annotated.

**Figure S18.** Manhattan plots for additional intestine-length and organ-weight traits. Intestine-length traits: (A) ileum length, (B) jejunum length, and (C) duodenum length. Organ-weight traits: (D) lung weight, (E) glandular stomach weight, and (F) muscular stomach weight. The red dashed line indicates the significance threshold (*p* < 1 × 10⁻⁵), and candidate genes at significant signals are annotated.

**Figure S19.** Enrichment of GWAS signals within gene and OCR modules. (A) Permutation test (B=1000) evaluating the global significance of trait-module enrichment. The histogram represents the null distribution of the number of significant pairs (*p* < 0.05) under randomized gene/OCR-module assignments for gene modules (left) and OCR modules (right). The red dashed line marks the observed number of significant module–trait pairs (empirical permutation *p* < 0.05). (B) The heatmap displays associations between 20 carcass traits (rows) and gene (g) or OCR (p) modules (columns); top annotations indicate module type and meta-module assignment. Liver weight-associated modules (g17 and p7), heart weight-associated modules (g28 and p11), and cecum length-associated modules (g31, g69, and p1) are highlighted. Significance codes indicate nominal *p* values from hypergeometric tests: *, *p* < 0.05; **, *p* < 0.01; ***, *p* < 0.001.

**Figure S20.** Tissue-specific activity of trait-associated gene and OCR modules. (A) Relative ATAC-seq signal distributions for p1, p7, and p11 and relative gene-expression distributions for g17, g28, g31, and g69 across tissues in the multi-tissue atlas. (B) Distribution of median gene expression for the corresponding g-modules across ChickenGTEx tissues. For box plots, center lines indicate medians, boxes indicate the interquartile range, and whiskers extend to 1.5 times the interquartile range.

**Figure S21.** Gene Ontology enrichment of trait-associated gene modules. The top ten enriched Gene Ontology biological-process terms are shown for each of the selected gene modules g17, g24, g28, and g31. Dot size represents the number of genes associated with each term, and color represents −log₁₀(FDR).

**Figure S22.** Motif enrichment and transcription factor activity across trait-associated p-modules. (A) Hierarchical clustering of 144 transcription factors (TFs) and their motifs based on sequence similarity, with the corresponding motif logos shown. (B) Enrichment of TF motifs across trait-associated p-modules. Orange cells indicate significant enrichment at −log₁₀(FDR) > 5. (C) Mean expression distributions across ChickenGTEx tissues for TFs enriched in the indicated p-modules. For box plots, center lines indicate medians, boxes indicate the interquartile range, and whiskers extend to 1.5 times the interquartile range.

**Figure S23.** Global transcription factor–target regulon network. The network contains 43 regulons comprising 454 nodes, with each regulon consisting of a transcription factor (TF) and its candidate downstream genes. Nodes are shown as pie charts indicating relative expression in liver (yellow) and heart (green), and directed edges connect TFs to their candidate targets. Selected TF-centered regulons are highlighted with red outlines.

# Supplementary Tables

**Table S1:** Information on the 44 chicken breeds included in this study.

**Table S2:** Sequencing and mapping statistics of high-depth WGS samples generated in this study and publicly available samples re-mapped in this work.

**Table S3:** List of candidate genes putatively under selection.

**Table S4:** Phenotypic measurements of 20 carcass traits in the 443 individuals used for GWAS.

**Table S5:** Genome-wide significant SNPs identified across 20 carcass-related traits.

**Table S6:** Annotation of genome-wide significant SNPs.

**Table S7:** Raw hypergeometric test *p* for GWAS signal enrichment in the 52 enriched modules.
